# Radiochemotherapy in stage III/IV nonmetastatic squamous cell head and neck cancer

**DOI:** 10.1038/sj.bjc.6603076

**Published:** 2006-05-16

**Authors:** C Schäfer, B Dietl, O Kölbl

**Affiliations:** 1 Department of Radiooncology, University of Regensburg Hospital, F:-J -Strauss Allee 11, D-93053 Regensburg, Germany

**Sir,**

We read the recently published article by Soo *et al* (2005) with great interest. They compared adjuvant radiotherapy following surgery with combined radiochemotherapy for locally advanced head and neck cancer. They could find no significant difference in the 3-year disease-free survival rate between the two methods of treatment. As we felt that some important points were missing in their paper, we would like to discuss these issues in the following.

We give the authors great credit for releasing the data of a trial that was prematurely stopped due to the slow accrual rate before the intended recruitment of 200 patients was reached. Considering the small size of the sample and the frequent deviation from protocol, it is questionable whether the trial is in a position to detect a significant difference at all. Contrary to the presented conclusion, the reported data such as the 3-year disease-free survival rate, 50 *vs* 40%, create the strong impression of an inferior outcome as a result of their combined radiochemotherapy. Although this difference did not reach statistical significance, it becomes more critical when compared to established radiochemotherapy regimens. For example, in the case of a daily fractionated radiation, the total dose should not be less than 70 Gy (Adelstein *et al*, 2003). Therefore, the total dose of 66 Gy, which was used in the study must be viewed as insufficient. Even in the case of hyperfractionated accelerated radiochemotherapy in locally advanced head and neck cancer, the applied total dose should not fall below 70 Gy, as recently published (Budach *et al*, 2005).

The authors report a much higher rate of grade 3 toxicity of the radiochemotherapy in comparison to their adjuvant radiotherapy method. Normally, higher toxicity is connected with treatment interruptions and, ultimately, with longer overall treatment time. Furthermore, it is known that longer overall treatment time can cause a reduction in the survival rate as well as in local control (Dietl *et al*, 2005). Moreover, higher toxicity can decrease the patient's quality of life in general. The above-mentioned inferiority of the combined treatment modality could thus be caused by the higher rate of grade 3 toxicity.

The authors rightly describe the low accrual rate as the major problem of their study. Unfortunately, they did not identify the cause for the low accrural rate which, we presume, is rooted in the study design. They selected patients with resectable head and neck cancer for their trial, but it is highly probable that such patients prefer surgery. Therefore, most studies concerning radiochemotherapy of head and neck cancer deal with unresectable tumours (Soo *et al*, 2005). The authors thus owe their readers an explanation as to which criteria they applied in distinguishing resectable from unresectable tumours. They point out the higher rate of organ-perservation in the laryngeal/hypopharyngeal disease *vs* the rest (68 *vs* 30%). This could be understood to mean that the authors had second thoughts as to whether every resectable tumour is preferably treated by surgery rather than radiochemotherapy. Therefore, we would like to pose the question as to which T4 tumours (56% of all patients) should be operated, especially under consideration of quality of life and loss of organ function. Resectable laryngeal cancer can set an impressive example as a recent prospective study was able to show (Forastiere *et al*, 2003). It was found that radiotherapy with concurrent cisplatin should be considered standard care for patients desiring laryngeal preservation whose cancer was within the categories of disease studied in their trial, and a laryngectomy should be performed only as a salvage therapy. It was concluded that, in most patients with laryngeal cancer, the disease can be managed without a primary surgical approach.

To make a long story short, we see two negative consequences of the applied study design, and the randomisation of resectable tumours. One consequence is the low accrual rate and the other is performance of surgery on patients who do not require such an intervention, as in the case of laryngeal cancer. The authors could not recognise this fact since they did not analyse quality of life among their patients. The abandonment of surgery can often be justified by quality of life considerations, as in the case of organ preservation. Regrettably, quality of life parameters were not included in this study.
